# Big Five Personality Traits Predict Successful Transitions From School to Vocational Education and Training: A Large-Scale Study

**DOI:** 10.3389/fpsyg.2020.01827

**Published:** 2020-07-31

**Authors:** Désirée Nießen, Daniel Danner, Marion Spengler, Clemens M. Lechner

**Affiliations:** ^1^GESIS – Leibniz Institute for the Social Sciences, Mannheim, Germany; ^2^University of Applied Labour Studies, Mannheim, Germany; ^3^Hector Research Institute of Education Sciences and Psychology, University of Tübingen, Tübingen, Germany

**Keywords:** personality, Big Five, socio-emotional skills, non-cognitive skills, educational transitions, school-to-work transition, vocational education and training

## Abstract

Educational transitions play a pivotal role in shaping educational careers, and ultimately social inequality. Whereas parental socioeconomic status (SES) and cognitive ability have long been identified as key determinants of successful educational transitions, much less is known about the role of socio-emotional skills. To address this gap, the present study investigated whether Big Five personality traits predict success in the transition from secondary school to vocational education and training (VET) above and beyond SES, cognitive ability, and other covariates. Using data from Starting Cohort 4 of the German National Educational Panel Study (NEPS; *N* = 4,137), we defined seven indicators of successful transition: obtaining a VET position, number of acceptances for VET positions, starting a VET position, (the absence of) dropout intentions and actual dropout, final VET grade, and satisfaction with VET. The results revealed that some Big Five traits were incrementally associated with several indicators of transition success. Conscientiousness emerged as the single most relevant trait, predicting all the transition success indicators but 1 (dropout intentions). The other Big Five traits had much weaker and less consistent links with transition success. Extraversion predicted the final VET grade and obtaining a VET position; Agreeableness was linked to a higher risk of dropout. Openness and Emotional Stability had no incremental effects on transition success. There was also some evidence for both compensatory and synergistic interactive effects, with Openness moderating mainly the effects of parental SES (on dropout intentions, actual dropout, and number of acceptances), and Agreeableness moderating the effects of cognitive ability (on obtaining a VET position, number of acceptances, and satisfaction with VET). Although individual effect sizes were small, the Big Five’s joint contribution to transition success was non-negligible, and often larger than that of sociodemographic characteristics and cognitive ability. Our results suggest a hitherto underappreciated contribution of personality to successful transitions to VET.

## Introduction

Throughout their educational careers, individuals are faced with various transitions, such as the transition from primary to secondary school or – in the German context – from secondary school to vocational education and training (VET) or higher education. By sorting individuals into different educational trajectories, educational transitions enable or constrain the range of possible options available to individuals in the future (Blossfeld et al., 2019; Erikson, 2019; Kogan, 2019; Stocké, 2019). By virtue of this, educational transitions play a pivotal role in shaping individuals’ long-term educational attainment, career prospects, and a range of associated outcomes such as health and well-being (Maaz et al., 2006; Klein et al., 2009; Schoon and Silbereisen, 2009).

Given the long-term consequences of educational transitions, it is important to understand why some individuals master such transitions successfully, whereas others do less well. Hence, successful transitions can be seen as an additional aspect of educational success, next to educational attainment and achievement. In this regard, previous research has identified several sources of individual differences in transition success, although their individual contributions are often small: sociodemographic characteristics (e.g., Blossfeld and Shavit, 1993; Duncan and Brooks-Gunn, 1997; Becker and Schubert, 2011); contextual factors such as social relationships or learning environments (e.g., Griebel and Niesel, 2004; Griebel, 2011); and cognitive ability (e.g., Gustafsson and Undheim, 1996). In contrast, little is known about the role of so-called socio-emotional (or “non-cognitive”) skills such as the Big Five personality traits (Costa and McCrae, 1992; Goldberg, 1992) in shaping educational transitions. Although a growing body of evidence attests to the incremental predictive validity of the Big Five for educational achievement and attainment (for a recent review, see Lechner et al., 2019), empirical studies on the role of socio-emotional skills in the success of educational *transitions* are almost absent from the literature.

Therefore, the question we addressed in the present study was: Can personality traits – understood as a proxy of socio-emotional skills – add to our understanding of why some individuals master educational transitions better than others? To address this question, we investigated whether the Big Five personality traits predict success in the transition from secondary school to VET in the German context. For this purpose, we leveraged data from a large-scale German panel study in which ninth-grade students were followed across the transition to VET. The German “dual system” of vocational education and training combines schooling with an apprenticeship (i.e., on-the-job training) at a company. Over the course of 2–3 years, apprentices spend part of their time at a company, where they get extensive training in a specific occupation, and the other part at a vocational school, where they receive education in occupation-related subjects (Heckhausen and Tomasik, 2002).

## Established Predictors of Educational Achievement and Successful Transitions

Previous research has identified several determinants and correlates of educational achievement and attainment, including successful transitions. Sociological research has focused mainly on the role of parental SES, gender, and migration background in predicting educational success (e.g., Blossfeld and Shavit, 1993; Duncan and Brooks-Gunn, 1997; Klein et al., 2009; Schoon, 2010; Becker and Schubert, 2011; Paat, 2015; McElvany et al., 2018). Psychological research has highlighted the crucial role of cognitive ability in shaping learning, and ultimately achievement and attainment (e.g., Kuncel et al., 2004; Deary et al., 2007; Roth et al., 2015). Research in educational science has focused additionally on the role of contextual factors such as social relationships or learning environments (e.g., Griebel and Niesel, 2004; Griebel, 2011). Among these determinants, sociodemographic characteristics and cognitive ability have typically shown the strongest links to educational success.

Although these predictors explain individual differences in educational achievement (e.g., grades) and attainment (e.g., the highest educational qualification obtained), their predictive power vis-à-vis educational *transitions* is limited. Thus, our understanding of transition success remains incomplete. Another important consideration is the nature of these predictors: parental SES and cognitive ability can hardly be changed. From a policy and intervention perspective, it would therefore be desirable to identify more malleable factors that contribute to successful educational transitions and that could be targeted by programs aimed at helping young people to master educational transitions. Here, we propose that socio-emotional skills – in particular the Big Five personality traits – might add to our understanding of transition success over and above the aforementioned established predictors.

## Personality Traits as Predictors of Educational Achievement

“Socio-emotional skills” is an umbrella term used to denote a broad set of individual difference constructs such as personality traits, motivation, and values. The common denominator of these constructs is that they refer to relatively consistent patterns of behavior, cognition, and affect that – although having a genetic basis – can be influenced by socialization and learning/experience, develop in interaction between environmental influences and biological predispositions, cannot easily be acquired, and have beneficial effects on relevant educational and life outcomes (see De Fruyt et al., 2015; Abrahams et al., 2019; Lechner et al., 2019). The Big Five framework (Costa and McCrae, 1992; Goldberg, 1992) is currently the most established and well-validated model of personality traits and is often used as a guiding framework in studies on socio-emotional skills. The framework comprises five global dimensions: Extraversion, Agreeableness, Conscientiousness, Emotional Stability, and Openness to Experience (henceforth only called Openness).

There is already a growing body of evidence pointing to incremental associations of the Big Five with success at school and at college/university as well as with career success – often over and above parental socioeconomic status (SES) and cognitive ability. Several meta-analyses (e.g., Poropat, 2009; Vedel and Poropat, 2017) and recent (large-scale) studies (e.g., Spengler et al., 2013, 2016; Lechner et al., 2017; Bergold and Steinmayr, 2018; Brandt et al., 2020) have identified Conscientiousness and Openness as the personality traits most relevant to educational achievement and attainment in both secondary and tertiary level students. Some of these studies (Spengler et al., 2016; Lechner et al., 2017; Brandt et al., 2020) showed that Conscientiousness was positively related to school grades and achievement test scores even when cognitive ability was controlled for. Similarly, Poropat’s (2009) meta-analysis of personality–academic performance relationships based on the Big Five model demonstrated that the effect sizes of Conscientiousness for school achievement [assessed by grades and grade point average (GPA)] rivaled that of cognitive ability and were incremental over – and independent of – cognitive ability. Recently, Diedrich et al. (2018) showed that Conscientiousness was the most robust positive predictor of GPA (achievement) – also specifically among VET students. Rammstedt et al. (2017) demonstrated a positive relationship between Conscientiousness and level of education (i.e., attainment). Openness was found to have positive but small associations with GPA and grades (i.e., achievement; Spengler et al., 2016) as well as with achievement test scores (Spengler et al., 2013) at school and university (Trapmann et al., 2007; Richardson et al., 2012; Vedel, 2014). Findings from a study on life outcomes identified a positive relationship between Openness and level of education (i.e., attainment; Rammstedt et al., 2017).

Agreeableness, Emotional Stability, and Extraversion have been found to have weaker and more inconsistent associations with educational and academic performance (e.g., Caspi et al., 2005; Poropat, 2009; Lechner et al., 2017; Vedel and Poropat, 2017). Agreeableness has been shown to have positive but small associations with GPA/grades (achievement; Poropat, 2009; Richardson et al., 2012; Vedel, 2014) and a positive relationship with life satisfaction (Rammstedt et al., 2017). Regarding Emotional Stability, Rammstedt et al. (2017) identified a positive relationship with level of education (attainment) and with life satisfaction. Concerning academic satisfaction, results of Trapmann et al. (2007) indicate a positive association with Emotional Stability. Finally, Rammstedt et al. (2017) found a negative relationship between Extraversion and the highest level of education (attainment). In sum, the Big Five have repeatedly replicated robust effects on a range of educational outcomes, above and beyond parental SES and cognitive ability.

## Personality Traits as Predictors of Successful Transitions

Are the Big Five personality traits as potent in predicting transition success as they are in predicting educational achievement and attainment? Extant findings refer only to a narrow set of global success indicators (such as GPA or the highest level of educational attainment; see above) and, in part, only to a priori selected personality traits such as Conscientiousness (Libbrecht et al., 2014; Shanahan et al., 2014b) or Extraversion and Neuroticism (Vasileva-Stojanovska et al., 2015). Other aspects of the transition process that constitute success, such as obtaining a VET position in the first place, have been neglected to date. This dearth of evidence may stem partly from the fact that there are few established indicators of successful transitions to VET – an issue that we addressed in the present study by operationalizing success in the transition to VET in a comprehensive fashion (see next section).

There is good reason to expect that the Big Five personality traits can contribute to transition success. We theorize that there are 2 principal pathways through which socio-emotional skills such as the Big Five personality traits might influence the success of educational transitions. Both of these pathways draw on an integrative social-ecological developmental model of agency that investigates the interplay of agency and structure in school-to-work transitions and the multiple influences shaping these transitions (Schoon and Heckhausen, 2019). The first pathway is individuals’ behavior during the transition process. Personality traits are psychosocial and self-regulatory resources (i.e., skills) that can be harnessed to select and pursue goals. In other words, they might foster individual agency during educational transitions (Heckhausen et al., 2010; DeYoung, 2013; Lechner et al., 2019). According to Schoon and Heckhausen (2019), “individual agency is most needed at times of transition, when individuals leave a pre-structured path.” Individual agency depends on resources such as the Big Five personality traits, which can therefore be regarded as prerequisites for agency, or, in economic terms, as human capital. For example, Extraversion and Agreeableness might help young people to build social relationships that can be instrumental in finding a VET position, and Conscientiousness might help them to prepare good application documents. Employers’ perception of VET applicants is the second pathway through which personality traits might act. It is likely that desirable and undesirable Big Five personality traits will be perceived by employers during the selection process and consequently rewarded or punished. For example, employers may choose VET applicants whom they perceive to be especially conscientious (e.g., Dunn et al., 1995; Caldwell and Burger, 1998; Moy and Lam, 2004), or they may prefer candidates who are emotionally stable (e.g., Dunn et al., 1995; Caldwell and Burger, 1998). We assume that the 2 pathways – individuals’ behaviors and employers’ perceptions – are inextricably linked, and that they contribute to transition success in complementary ways.

Irrespective of the specific pathways through which personality traits may affect transition success, their associations with indicators of transition success can take 2 main forms: additive and interactive. An additive effect (or “main effect”) would mean that personality has an incremental linear association with transition success above and beyond the effects of other predictors, such as parental SES and cognitive ability.

An interactive effect (or “moderation effect”) would imply that the strength of other predictors, such as parental SES or cognitive ability, varies in dependence on personality. Such interactive effects may be compensatory or synergistic in nature. According to resource substitution theory (Mirowsky and Ross, 2003), low resources in 1 domain can be substituted by resources in another domain. For example, cognitive ability may be more important for individuals with low SES, because high cognitive ability can compensate for low SES. This suggests that personality traits such as Conscientiousness may also be able to compensate for low SES or low cognitive ability. In contrast, a synergistic interactive effect is an effect where high resources in 1 domain augment the effect of resources in another domain. According to Damian et al.’s (2015) Matthew effect hypothesis (the Matthew effect was originally operationalized by Merton, 1968), personality traits are more relevant under advantaged developmental conditions such as a higher level of parental SES.

Only a few studies have tested possible interactive effects of personality with SES and cognitive ability. For example, Sackett et al. (1998) and Danner et al. (2019) found interactions between personality and sociodemographic factors in predicting job performance. Even fewer studies have tested such compensatory or synergistic effects with regard to educational outcomes (e.g., Shanahan et al., 2014a; Damian et al., 2015; Rammstedt et al., 2016; Ayoub et al., 2018; Bergold and Steinmayr, 2018). For example, with regard to the prediction of educational attainment, Ayoub et al. (2018) reported a compensatory interactive effect between parental SES and Emotional Stability, Openness, Conscientiousness, and Agreeableness; Damian et al. (2015) reported a compensatory interactive effect between parental SES and Conscientiousness, Agreeableness, and Extraversion; and Shanahan et al. (2014a) reported a compensatory interactive effect between parental SES and Agreeableness, Extraversion, Openness, and Emotional Stability. Results from Bergold and Steinmayr (2018) suggest positive interactive associations between cognitive ability and Conscientiousness and Emotional Stability in predicting senior secondary school GPA. Rammstedt et al. (2016) found a negative interactive effect between Conscientiousness and labor force participation and a positive interactive effect between Openness and educational attainment in predicting cognitive ability.

## Aims and Research Questions of the Present Investigation

In sum – despite empirical evidence of robust links between personality and educational success in general – it remains largely unclear whether the Big Five personality traits play a role in shaping educational transitions. To close this research gap, we investigated in the present study whether the Big Five personality traits predict success in the transition from lower secondary or intermediate secondary school to VET above and beyond parental SES, gender, migration background, and cognitive ability. Moreover, we aimed to identify the specific role of personality in shaping transition success by testing whether the Big Five show mainly additive associations with transition success (i.e., main effects), or whether they also moderate the effects of other established predictors of transition success, in particular parental SES and cognitive ability (i.e., interactive effects). Because there is little previous work to build on, the latter analyses of interactive effects are purely exploratory in nature. We comprehensively operationalized transition success with the following seven indicators (for details, see Measures): obtaining a VET position, number of acceptances, starting a VET position, (absence of) dropout intentions, (absence of) actual dropout, final VET grade, and satisfaction with VET.

Based on previous findings on how the Big Five contribute to educational achievement and attainment (e.g., Spengler et al., 2013, 2016; Lechner et al., 2019; Brandt et al., 2020), we expected Conscientiousness, Emotional Stability, Openness, and Extraversion to have consistently positive associations with all aspects of transition success above and beyond the effects of the covariates (additive effects). For Agreeableness, we had no specific expectation, and we examined its effects in an exploratory fashion. The rationale behind our expectations was as follows: We presumed that Conscientiousness would manifest itself in performance effort and application behavior in terms of the number and type of applications. In addition, Conscientiousness itself could be a criterion in the selection process. Emotional Stability could manifest itself in a better handling of demands and overextension. In addition, Emotional Stability could curb test anxiety or anxiety during the application procedure. We assumed that Openness would lead to more creative apprenticeship search strategies and to greater openness toward different sectors. Extraversion describes the tendency to engage in social behavior and could therefore be helpful for acquiring a social network. Furthermore, Extraversion could manifest itself in assertiveness in the application procedure. Agreeableness could also foster the development of a social network by being cooperative and compassionate. Additionally, Agreeableness could reflect sympathy, which appears to be beneficial in selection procedures. In contrast, low Agreeableness may be accompanied by high task orientation, which is also relevant to success.

We further expected that, in addition to having additive effects, personality traits would moderate the associations between established predictors of transition success – namely, parental SES and cognitive ability – and our seven success indicators. Given the lack of previous evidence and pertinent theorizing regarding possible interactions between personality and sociodemographic characteristics or cognitive ability, we refrained from formulating specific hypotheses in this regard. Instead, we tested these interactive effects in an exploratory fashion. We classified any interaction that emerged according to whether it was compensatory or synergistic in nature.

## Materials and Methods

### Database and Sample

We used data from the German National Educational Panel Study (NEPS): Starting Cohort 4 (Grade 9; Blossfeld and Roßbach, 2011; doi: 10.5157/NEPS:SC4:9.1.0). NEPS is an ongoing longitudinal multi-cohort panel study. Starting Cohort 4 comprises students who were attending ninth grade in the 2010/2011 school year. Students from this cohort were first interviewed in autumn/winter 2010/2011 (wave 1), when they were in ninth grade. They were re-interviewed biannually until spring 2013 (waves 2–6) and annually thereafter until autumn 2015/spring 2016 (waves 7–9). The survey mode varied between paper-and-pencil interviewing (PAPI) for students and computer-assisted telephone/personal interviewing (CATI/CAPI) for school-leavers. For the present research, we used data from waves 1 to 7. For every individual, information on the variables was assessed once. Information on personality traits, sociodemographic variables, and cognitive ability was gathered in grade 9 (waves 1–2) before the transition from school to VET. Information on the success indicators was gathered within waves 3–7.

Germany has a very stratified school and vocational training system. After primary school, students are selected into different school types: *Hauptschule* (school at lower secondary level providing a basic secondary education), *Realschule* (intermediate secondary school), and *Gymnasium* (academically oriented secondary schools or school tracks). Graduates from *Hauptschule* leave the school system after 9th grade at the age of 15, graduates from *Realschule* after 10th grade at the age of 16, and graduates from *Gymnasium* after 12th or 13th grade at the age of 18 or 19 with different levels of school-leaving certificates. Graduates from *Hauptschule* and *Realschule* are eligible to do a VET, while graduates from *Gymnasium* have the possibility to go to college/university^1^. In addition to these three “regular” school types, there are so-called *Förderschulen* (special needs schools), which students with disabilities, such as learning, physical, or developmental disabilities, attend.

Beginning with *N* = 16,425 participants, we reduced the sample to individuals who had graduated from *Hauptschule* after 9th grade or from *Realschule* after 10th grade, and for whom data were available since wave 1 (*n* = 16,052). The reason why we only investigated the transition from school to VET was that the dataset simply did not allow investigating other transitions. We excluded students from *Gymnasium* because no student from this school type in the sample transitioned to VET during the observation period (*n* = 5,568). We also excluded students from *Förderschulen* (*n* = 1,186) because they cannot be compared to students from “regular” schools and students from Waldorf schools (*n* = 171) because these schools are based on a completely different pedagogical principle compared to “regular” schools without, for instance, grading or grade retention. We also excluded students with wave-specific temporary or final dropouts (e.g., no data available since graduation or individual tracking no longer possible; *n* = 3,556); students whose first vocational track did not begin until Wave 8 or 9 (*n* = 530); students with inconsistent spell data (e.g., because they entered a vocational preparation program [*Berufsvorbereitung*] or underwent vocational training prior to graduation; *n* = 311); and students with missings on the Big Five questionnaire (*n* = 416). This resulted in a total of 4,314 school-leavers. The mean age of the students in the first wave was 15.3 years old (*SD* = 0.7; 42.4% female).

Most of these school-leavers (*N* = 4,137; 96%) applied for a VET position within the first year after graduation. The majority among them (*N* = 3,524; 85%) obtained an acceptance for a VET position; 68% (*N* = 2,411) of those who obtained an acceptance actually started VET within the first year after graduation.

### Measures

#### Big Five Personality Traits

The Big Five personality traits were assessed with the 10-item Big Five Inventory (BFI-10; Rammstedt and John, 2007) plus 1 additional item for the Agreeableness domain. The BFI-10(+1) is an established and widely used 10-item short scale with 2 items per dimension that is used, for example, in the World Value Survey (WVS) and in the International Social Survey Programme (ISSP) and has satisfying psychometric quality criteria (e.g., Rammstedt and John, 2007; Rammstedt et al., 2014). All 11 items were to be answered on a 5-point response scale ranging from *strongly disagree* (1) to *strongly agree* (5). In the present sample, internal consistency (as measured by the Spearman-Brown formula, which is appropriate for 2-item scales) for the Big Five dimensions ranged from 0.35 (Agreeableness) to 0.55 (Extraversion). These values are sufficient for 2-item scales because the items are designed to assess heterogeneous facets of the Big Five dimensions (Rammstedt and John, 2007). Importantly, previous research shows that the BFI-10(+1)’s test–retest reliabilities are much higher (on average *r* = 0.75; see Rammstedt and John, 2007) than its internal consistencies. Furthermore, the BFI-10(+1)’s predictive validity for a broad range of criteria is as high as – and sometimes higher than – that of much longer Big Five scales (Thalmayer et al., 2011). Because the BFI-10 is a balanced scale, the scale scores implicitly control for acquiescence. Therefore, we used the manifest scale scores (Big Five personality traits and covariates) as predictors, and we modeled the interactions between personality traits and covariates as multiplicative terms (as centered variables, except for migration background). Negatively keyed items were recoded beforehand.

#### Transition Success Indicators

There is no clear consensus in the literature on school-to-work transitions as to what constitutes a successful transition to VET. Consequently, to address our research questions, we first defined what constitutes a successful transition to VET and selected appropriate success indicators. Our criteria for selecting these success indicators were that the indicator should (a) be positively valued by individuals and society and (b) have long-term consequences for individuals’ further life chances. Thus, the indicators should capture a normative understanding of transition success from a life-course perspective. Moreover, (c) the indicators should refer to a critical phase of the transition from school to VET – namely, the initial phase (1 year after leaving school), the intermediate phase (1 year after starting a VET position), or the concluding phase (during VET). In line with these criteria, we selected the following seven success indicators in order to obtain a depiction of transition success as comprehensive as possible with the given data (NEPS dataset): (a) obtaining a VET position within 1 year after graduation (i.e., acceptance by an employer after the submission of an application); (b) number of acceptances for VET positions within 1 year after graduation; (c) starting a VET position within 1 year after graduation (given the receipt of an acceptance for a VET position); (d) (absence of) dropout intentions; (e) (absence of) actual dropout; (f) final VET grade; and (g) satisfaction with VET after 1 year in a VET position.

Obtaining a VET position was operationalized with *yes* (1) vs. *no* (0). The number of acceptances for VET positions was assessed with the question “How many acceptances did you get in all? Tell me the number of apprenticeships you were offered.” and ranged from 0 to 20. Starting a VET position was operationalized with *yes* (1) vs. *no* (0). Dropout intentions were assessed with the question “Are you seriously considering at this time changing or dropping out of your apprenticeship/vocational training program?” Possible answers were *yes* (1) or *no* (0). Actual dropout was measured with the question: “Did you end the vocational training early or did you stay to the end but not earn the qualification?” Possible answers were *yes* (1) or *no* (0). The final VET grade was measured with the question “What was your overall grade for this vocational training program?” and theoretical ranges – after recoding (7 – raw score) – from *low* (1) to *high* (6); in the present sample, the values ranged from *low* (2.8) to *high* (6.0). Satisfaction with VET was assessed with the question “How satisfied are you with your vocational training program?” on a scale ranging from *completely dissatisfied* (0) to *completely satisfied* (10).

#### Control Variables

We included the following established predictors of transition success as statistical control variables in order to investigate the incremental predictive power of personality traits: (a) parental SES [International Socio-Economic Index of Occupational Status (ISEI-08; Ganzeboom et al., 1992); ISEI describes the occupational status as both level of education needed for a specific occupation and the corresponding income of that specific occupation (Züll, 2015) ranging from *low* (11.56; i.e., farmers), to *high* (88.96; i.e., judges); it was assessed with the open question “What profession do your parents currently pursue? For example, car mechanic, shop assistant, teacher at a Gymnasium, civil engineer. If either your mother or father is currently not working, please think of her or his last professional activity.” and then assigned to different codings of standard categorization schemes of occupations, among others the ISEI – if a student’s parents had different values, we used the highest ISEI in the family]; (b) migration background (captured via the proxy of having German as a mother tongue; *yes* [1] vs. *no* [0]); (c) gender [*male* (1) vs. *female* (2)]; and (d) cognitive ability. Cognitive ability was assessed with the NEPS reasoning test (NEPS-MAT), a figural reasoning task that measures general cognitive ability with 12 items (see Pohl and Carstensen, 2012) ranging from *low* (0) to *high* (12). In the present sample, internal consistency (Cronbach’s alpha) was 0.66.

### Analysis

We examined the association between transition success and personality with OLS regression models for the quasi-continuous dependent variables (number of acceptances for VET positions, final VET grade, and satisfaction with VET) and logistic regressions for the dichotomous dependent variables [obtaining a VET position, starting a VET position, (absence of) dropout intentions, and (absence of) actual dropout]. To facilitate the interpretation of the results of the logistic regressions, we report the average marginal effects (AMEs). AMEs have a straightforward interpretation as probabilities.

In the first step, we analyzed the association between the Big Five traits and the seven indicators of transition success (Model I). In the second step, we added the covariates in order to examine whether the Big Five incrementally predicted transition success over and above these covariates (Model II). In the third and fourth steps, we additionally included interaction terms between the Big Five traits and 1 covariate at a time – cognitive ability in the third model, parental SES in the fourth model – in order to examine whether personality traits moderated the association between parental SES, cognitive ability, and success (Models III–IV). To keep the sample size within each dependent variable equivalent across the individual models (I–IV), we used complete case analysis and only analyzed data of students without missing values on the independent variables. The statistical analyses were run with Stata.

## Results

### Descriptive Statistics

Descriptive statistics for the personality traits, the covariates, and the success indicators are depicted in Table 1. As can be seen from that table, there was substantial variation in all variables. Table 2 shows the correlations between Big Five traits, the success indicators, and the covariates. As can be seen from that table, there were small associations between Extraversion, Emotional Stability, Openness, and in particular Conscientiousness and several success indicators (−0.10 ≤ *r* ≤ 0.08), suggesting that personality is related to at least some of our transition success indicators. Table 2 further reveals that the Big Five personality traits were moderately associated with cognitive ability (−0.10 ≤ *r* ≤ 0.06), parental SES (−0.08 ≤ *r* ≤ 0.06), migration background (*r* = 0.06), and gender (−0.21 ≤ *r* ≤ 0.19). We therefore used multiple regression analyses to examine whether the Big Five explained transition success above and beyond sociodemographic characteristics and cognitive ability.

**TABLE 1:** Descriptive statistics for continuous and categorical variables.

Continuous variables	*M*	*SD*	No. of items	Cronbach’s alpha	*N*
Extraversion	3.41	0.87	2	0.55	4,314
Agreeableness	3.46	0.68	3	0.34	4,314
Conscientiousness	3.20	0.87	2	0.46	4,314
Emotional stability	3.22	0.85	2	0.35	4,314
Openness	3.35	0.93	2	0.36	4,314
Cognitive ability	7.71	2.59	12	0.66	3,993
Parental SES	43.57	18.16	1		3,701
Number of acceptances	1.89	2.31	1		3,238
Final VET grade	2.53	0.65	1		954
Satisfaction with VET	8.17	1.53	1		1,811

**Categorical variables**	**Categories**				***n***

Obtaining a VET position	0: No				613
	1: Yes				3,524
Starting a VET position	0: No				1,113
	1: Yes				2,411
Dropout intentions	0: No				2,222
	1: Yes				118
Actual dropout	0: No				2,107
	1: Yes				304
Gender	1: Male				2,484
	2: Female				1,830
Migration background	0: German as mother tongue				3,779
	1: Other mother tongue(s)				490

*The Big Five scores range between 1 and 5 (strongly disagree–strongly agree); cognitive ability ranges between 0 and 12 (sum score); parental SES ranges between 11.56 and 88.96 (low–high); number of acceptances ranges between 0 and 20; final VET grade ranges between 0 and 4.2 (high–low); satisfaction with VET ranges between 0 and 10 (completely dissatisfied–completely satisfied).*

**TABLE 2:** Correlations between the Big Five personality traits and the success indicators and covariates.

		Extraversion			Agreeableness			Conscientiousness			Emotional Stability			Openness		
Success indicators	*N*	*r*	CI_95%_	*p*	*r*	CI_95%_	*p*	*r*	CI_95%_	*p*	*r*	CI_95%_	*P*	*r*	CI_95%_	*p*
Obtaining a VET position	4,137	**0.04**	[0.01, 0.07]	0.022	–0.01	[−0.04, 0.02]	0.624	**0.04**	[0.01, 0.07]	0.006	**0.03**	[0.00, 0.06]	0.046	–0.02	[−0.05, 0.01]	0.172
Number of acceptances	3,238	0.02	[−0.02, 0.05]	0.312	0.03	[−0.01, 0.06]	0.092	**0.06**	[0.02, 0.09]	0.001	0.02	[−0.01, 0.06]	0.162	–0.00	[−0.04, 0.03]	0.831
Starting a VET position	3,524	–0.01	[−0.04, 0.03]	0.698	–0.02	[−0.05, 0.02]	0.299	0.02	[−0.01, 0.06]	0.177	**0.04**	[0.00, 0.07]	0.036	–0.01	[−0.05, 0.02]	0.422
Dropout intentions	2,340	0.01	[−0.03, 0.06]	0.495	–0.03	[−0.07, 0.01]	0.177	–0.03	[−0.07, 0.01]	0.198	0.02	[−0.02, 0.06]	0.387	0.03	[−0.01, 0.07]	0.199
Actual dropout	2,411	0.03	[−0.01, 0.07]	0.211	0.03	[−0.01, 0.07]	0.184	**−0.05**	[−0.09, −0.01]	0.027	–0.01	[−0.05, 0.03]	0.752	0.03	[−0.01, 0.07]	0.135
Final VET grade	954	**0.10**	[0.04, 0.16]	0.002	–0.01	[−0.08, 0.05]	0.714	0.05	[−0.01, 0.11]	0.128	0.06	[−0.01, 0.12]	0.083	**0.09**	[0.02, 0.15]	0.009
Satisfaction with VET	1,811	–0.00	[−0.05, 0.05]	0.997	0.04	[−0.01, 0.09]	0.101	**0.08**	[0.03, 0.12]	0.001	**0.07**	[0.02, 0.11]	0.005	–0.04	[−0.09, 0.01]	0.089
**Covariates**																
Cognitive ability	3,993	**−0.06**	[−0.09, −0.03]	0.000	0.02	[−0.01, 0.06]	0.134	**−0.10**	[−0.13, −0.07]	0.000	**0.05**	[0.02, 0.08]	0.002	**0.06**	[0.03, 0.09]	0.000
Parental SES	3,701	**0.04**	[0.01, 0.07]	0.010	**−0.05**	[−0.08, 0.01]	0.005	**−0.08**	[−0.11, 0.04]	0.000	**0.05**	[0.01, 0.08]	0.005	**0.06**	[0.02, 0.09]	0.001
Gender	4,314	0.02	[−0.01, 0.05]	0.235	**0.12**	[0.09, 0.15]	0.000	**0.19**	[0.16, 0.22]	0.000	**−0.21**	[−0.24, 0.19,]	0.000	**0.17**	[0.14, 0.20]	0.000
Migration background	4,269	–0.01	[−0.04, 0.02]	0.631	0.01	[−0.02, 0.04]	0.433	**0.06**	[0.03, 0.09]	0.000	–0.02	[−0.05, 0.01]	0.290	0.03	[−0.01, 0.06]	0.099

*Coefficients significant at the p < 0.05 level are in bold type.*

### Multivariate Models Predicting Successful Transitions

The regression estimators for the seven success indicators are displayed in Tables 3–9 (unstandardized coefficients; *b* for quasi-continuous outcomes; AMEs for dichotomous outcomes). Statistically significant interactions are additionally depicted in Supplementary Figures S1–S6. We report only statistically significant effects (*p* < 0.05) in the text.

**TABLE 3:** Average marginal effects for obtaining a VET position within 1 year after graduation (given the submission of an application) regressed on the Big Five and the covariates.

Model	I			II			III			IV		
	AME	CI_95%_	*p*	AME	CI_95%_	*p*	AME	CI_95%_	*p*	AME	CI_95%_	*p*
C	**0.017**	[0.003, 0.031]	0.018	**0.027**	[0.012, 0.041]	0.000	**0.028**	[0.014, 0.043]	0.000	**0.028**	[0.014, 0.042]	0.000
ES	0.008	[−0.006, 0.022]	0.256	–0.001	[−0.016, 0.013]	0.862	–0.001	[−0.015, 0.014]	0.933	0.000	[−0.014, 0.015]	0.965
O	–0.010	[−0.023, 0.002]	0.115	–0.008	[−0.021, 0.005]	0.224	–0.007	[−0.020, 0.005]	0.255	–0.009	[−0.022, 0.004]	0.173
E	**0.015**	[0.001, 0.028]	0.037	**0.017**	[0.004, 0.031]	0.014	**0.018**	[0.004, 0.031]	0.012	**0.018**	[0.004, 0.032]	0.010
A	–0.007	[−0.025, 0.011]	0.460	–0.004	[−0.022, 0.014]	0.633	–0.008	[−0.027, 0.010]	0.368	–0.005	[−0.023, 0.013]	0.605
Cognitive ability				**0.008**	[0.003, 0.012]	0.001	**0.008**	[0.004, 0.013]	0.000	**0.008**	[0.003, 0.012]	0.001
Parental SES				0.001	[−0.000, 0.001]	0.051	**0.001**	[0.000, 0.001]	0.044	**0.001**	[0.000, 0.002]	0.012
Gender				**−0.046**	[−0.070, −0.021]	0.000	**−0.047**	[−0.071, −0.023]	0.000	**−0.048**	[−0.072, −0.023]	0.000
Migration background				**−0.073**	[−0.106, −0.039]	0.000	**−0.072**	[−0.106, −0.038]	0.000	**−0.074**	[−0.107, −0.040]	0.000
Cognitive ability × C							0.004	[−0.001, 0.009]	0.157			
Cognitive ability × ES							0.002	[−0.003, 0.007]	0.406			
Cognitive ability × E							0.004	[−0.001, 0.009]	0.161			
Cognitive ability × O							0.001	[−0.004, 0.006]	0.680			
Cognitive ability × A							**−0.008**	[−0.015, −0.001]	0.018			
SES × C										0.001	[−0.000, 0.001]	0.193
SES × ES										**0.001**	[0.000, 0.002]	0.003
SES × O										0.000	[−0.001, 0.001]	0.980
SES × E										0.000	[−0.000, 0.001]	0.321
SES × A										–0.001	[−0.002, 0.000]	0.152
*Pseudo R*^2^	**0.006**		0.008	**0.028**		0.000	**0.033**		0.000	**0.034**		0.000

*Model I: Big Five only; Model II: Big Five and covariates; Model III: Big Five, covariates, and interaction terms between the Big Five and cognitive ability; Model IV: Big Five, covariates, and interaction terms between the Big Five and parental SES. C: Conscientiousness; ES: Emotional Stability; O: Openness; E: Extraversion: A: Agreeableness. N = 3,276. Coefficients and R^2^ significant at the p < 0.05 level are in bold type.*

#### Obtaining a VET Position

Our first success indicator was obtaining a VET position within 1 year after graduation (given the submission of an application). As shown in Table 3, high Conscientiousness was associated with a 1.7% higher likelihood of obtaining a VET position, and high Extraversion was associated with a 1.5% higher likelihood. Overall, personality explained 0.6% of the variance (*Pseudo R*^2^; Model I). Analyzing the effects of personality traits and covariates simultaneously, Model II explained 2.8% of the overall variance (*Pseudo R*^2^) and indicated a significant association between high cognitive ability (0.8% higher likelihood), being male (4.6% higher likelihood), not having a migration background (7.3% higher likelihood), and obtaining a VET position. Nevertheless, Model II revealed that the effects of Conscientiousness (2.7% higher likelihood) and Extraversion (1.7% higher likelihood) were even greater compared to Model I, and that they were incremental. The maximum difference between a student scoring at the lowest possible value of Conscientiousness (i.e., 1 on the 5-point scale) and the highest possible value (i.e., 5 on the 5-point scale) was (5–1)^∗^2.7% = 10.8%, which is larger than that of all significant covariates. The maximum difference in the case of Extraversion was 6.8%, which was therefore larger than that of gender, but somewhat smaller than that of cognitive ability (9.6%) and migration background.

Supplementary Figure S1 illustrates that 2 Big Five personality traits interacted with different covariates. First, low Agreeableness was more detrimental for students with low cognitive ability, whereas it was helpful for students with high cognitive ability (0.8%; Model III). Second, Emotional Stability was more detrimental for students with low parental SES and more helpful for students with high parental SES (0.1%; Model IV).

#### Number of Acceptances for VET Positions

The second success indicator was the number of acceptances for VET positions within 1 year after graduation. Table 4 indicates that Conscientiousness was positively associated with the number of acceptances for VET positions (*b* = 0.109), even when adjusted for the covariates (*b* = 0.129), of which only gender was associated with the number of acceptances (with males obtaining more acceptances compared to females; *b* = −0.215). Personality alone explained 0.3% of the overall variance (Model I); personality and covariates together explained 0.5% of the overall variance (Model II). Even though the models are not statistically significant overall, it is noteworthy that the effect of Conscientiousness increased over Model I. Furthermore, after standardizing the variable, it became apparent that Conscientiousness (*b*_std_ = 0.516) was even more predictive than gender.

**TABLE 4:** Unstandardized regression coefficients for the number of acceptances for VET positions within 1 year after graduation regressed on the Big Five and the covariates.

Model	I			II			III			IV		
	*b*	CI_95%_	*p*	*b*	CI_95%_	*p*	*b*	CI_95%_	*p*	*b*	CI_95%_	*p*
C	**0.109**	[0.002, 0.217]	0.047	**0.129**	[0.019, 0.239]	0.022	**0.127**	[0.017, 0.237]	0.024	**0.130**	[0.020, 0.240]	0.021
ES	0.052	[−0.057, 0.162]	0.350	0.022	[−0.091, 0.135]	0.705	0.020	[−0.093, 0.133]	0.724	0.020	[−0.094, 0.133]	0.735
O	–0.012	[−0.111, 0.086]	0.805	0.006	[−0.095, 0.106]	0.913	0.010	[−0.091, 0.111]	0.844	0.002	[−0.098, 0.103]	0.962
E	0.045	[−0.060, 0.151]	0.401	0.055	[−0.052, 0.161]	0.314	0.049	[−0.058, 0.155]	0.372	0.054	[−0.053, 0.160]	0.321
A	0.058	[−0.081, 0.197]	0.413	0.072	[−0.068, 0.212]	0.311	0.068	[−0.071, 0.208]	0.338	0.067	[−0.073, 0.206]	0.351
Cognitive ability				0.001	[−0.034, 0.037]	0.950	0.001	[−0.034, 0.037]	0.936	0.001	[−0.034, 0.037]	0.948
Parental SES				0.000	[−0.005, 0.005]	0.911	0.000	[−0.005, 0.006]	0.863	0.000	[−0.005, 0.005]	0.885
Gender				**−0.215**	[−0.408, −0.022]	0.029	**−0.220**	[−0.413, −0.027]	0.026	**−0.210**	[−0.403, −0.017]	0.033
Migration background				–0.043	[−0.375, 0.289]	0.799	–0.038	[−0.370, 0.294]	0.822	–0.040	[−0.372, 0.292]	0.815
Cognitive ability × C							0.020	[−0.023, 0.063]	0.364			
Cognitive ability × ES							0.001	[−0.042, 0.044]	0.955			
Cognitive ability × E							0.013	[−0.028, 0.055]	0.530			
Cognitive ability × O							–0.033	[−0.072, 0.006]	0.098			
Cognitive ability × A							**−0.056**	[−0.112, −0.001]	0.048			
SES × C										**−0.006**	[−0.012, −0.000]	0.049
SES × ES										–0.002	[−0.008, 0.004]	0.472
SES × O										**0.006**	[0.000, 0.011]	0.037
SES × E										0.004	[−0.002, 0.010]	0.208
SES × A										0.001	[−0.007, 0.009]	0.807
*R*^2^	0.003		0.162	0.005		0.169	0.008		0.089	0.009		0.073

*Model I: Big Five only; Model II: Big Five and covariates; Model III: Big Five, covariates, and interaction terms between the Big Five and cognitive ability; Model IV: Big Five, covariates, and interaction terms between the Big Five and parental SES. C: Conscientiousness; ES: Emotional Stability; O: Openness; E: Extraversion: A: Agreeableness. N = 2,606. Coefficients and R^2^ significant at the p < 0.05 level are in bold type.*

As can be seen from Supplementary Figure S2, Agreeableness compensated for low cognitive ability (*b* = −0.056; Model III). In addition, high Conscientiousness (*b* = −0.006) and low Openness (*b* = 0.006) led to a higher number of acceptances for VET positions when parental SES was low (Model IV).

#### Starting a VET Position

The third success indicator was starting a VET position within 1 year after graduation (given the receipt of an acceptance for a VET position). As can be seen in Table 5, high Conscientiousness was associated with a 2.2% higher likelihood of starting a VET position; high Emotional Stability was associated with a 2.6% higher likelihood; and low (not high) Agreeableness was associated with a 2.8% higher likelihood (Model I). Personality traits explained 0.4% of the overall variance (*Pseudo R*^2^; Model I). Incorporating personality traits and covariates jointly into the model (Model II), we found that the pattern of significant predictors changed. The positive effect of Conscientiousness increased (3.2% higher likelihood) and was incremental; the relationship with Emotional Stability and Agreeableness disappeared. In total, Model II explained 1.5% of the overall variance (*Pseudo R*^2^). Furthermore, there was a positive association between cognitive ability (0.8% higher likelihood), parental SES (0.1% higher likelihood), and not having a migration background (13.4% higher likelihood) and starting a VET position. After standardizing the variables, Model II indicated that Conscientiousness – with a 12.8% higher likelihood – had a larger effect on starting a VET position than three of the four established predictors. Only migration background still had slightly more predictive power (cognitive ability: 9.6%; SES: 7.7%).

**TABLE 5:** Average marginal effects for starting a VET position within 1 year after graduation (given the receipt of an acceptance for a VET position) regressed on the Big Five and the covariates.

Model	I			II			III			IV		
	AME	CI_95%_	*p*	AME	CI_95%_	*p*	AME	CI_95%_	*p*	AME	CI_95%_	*p*
C	**0.022**	[0.002, 0.042]	0.034	**0.032**	[0.011, 0.052]	0.002	**0.032**	[0.012, 0.053]	0.002	**0.032**	[0.011, 0.052]	0.002
ES	**0.026**	[0.005, 0.047]	0.014	0.017	[−0.005, 0.038]	0.124	0.017	[−0.004, 0.038]	0.122	0.016	[−0.005, 0.038]	0.132
O	–0.004	[−0.022, 0.015]	0.696	–0.002	[−0.021, 0.017]	0.841	–0.002	[−0.020, 0.017]	0.858	–0.002	[−0.021, 0.016]	0.794
E	–0.017	[−0.037, 0.004]	0.107	–0.015	[−0.036, 0.005]	0.133	–0.015	[−0.035, 0.005]	0.137	–0.015	[−0.035, 0.005]	0.136
A	**−0.028**	[−0.055, −0.002]	0.036	–0.025	[−0.052, 0.001]	0.060	–0.025	[−0.051, 0.001]	0.064	–0.025	[−0.051, 0.001]	0.065
Cognitive ability				**0.008**	[0.001, 0.015]	0.020	**0.008**	[0.001, 0.015]	0.020	**0.008**	[0.002, 0.015]	0.016
Parental SES				**0.001**	[0.000, 0.002]	0.042	**0.001**	[0.000, 0.002]	0.020	**0.001**	[0.000, 0.002]	0.049
Gender				–0.034	[−0.070, 0.002]	0.067	–0.034	[−0.070, 0.002]	0.062	–0.031	[−0.067, 0.004]	0.086
Migration background				**−0.134**	[−0.192, −0.077]	0.000	**−0.134**	[−0.191, −0.076]	0.000	**−0.130**	[−0.188, −0.073]	0.000
Cognitive ability × C							0.002	[−0.006, 0.010]	0.630			
Cognitive ability × ES							0.004	[−0.004, 0.012]	0.335			
Cognitive ability × E							–0.001	[−0.009, 0.007]	0.859			
Cognitive ability × O							0.003	[−0.005, 0.010]	0.486			
Cognitive ability × A							–0.000	[−0.011, 0.010]	0.949			
SES × C										0.000	[−0.001, 0.001]	0.603
SES × ES										**−0.001**	[−0.002, −0.000]	0.036
SES × O										0.000	[−0.001, 0.001]	0.490
SES × E										**0.002**	[0.000, 0.003]	0.002
SES × A										0.001	[−0.000, 0.002]	0.159
*Pseudo R*^2^	**0.004**		0.016	**0.015**		0.000	**0.016**		0.000	**0.019**		0.000

*Model I: Big Five only; Model II: Big Five and covariates; Model III: Big Five, covariates, and interaction terms between the Big Five and cognitive ability; Model IV: Big Five, covariates, and interaction terms between the Big Five and parental SES. C: Conscientiousness; ES: Emotional Stability; O: Openness; E: Extraversion: A: Agreeableness. N = 2,846. Coefficients and R^2^ significant at the p < 0.05 level are in bold type.*

Supplementary Figure S3 demonstrates that both Emotional Stability (0.1%) and Introversion (0.2%) compensated for low parental SES (Model IV) in the prediction of starting a VET position.

#### Dropout Intentions

The fourth success indicator was (the absence of) dropout intentions. As can be seen in Table 6, this outcome variable was positively related to Openness (1.1%). However, the model was not significant, with an overall explained variance of 0.9% (*Pseudo R*^2^; Model I). Considering both personality traits and covariates simultaneously in Model II, we found that the positive association with Openness vanished. In total, Model II explained 2.9% of the overall variance (*Pseudo R*^2^). In addition, being female (2.9% higher likelihood) and having a migration background (4.2% higher likelihood) were positively associated with the intentions of dropping out of VET.

**TABLE 6:** Average marginal effects for dropout intentions regressed on the Big Five and the covariates.

Model	I			II			III			IV		
	AME	CI_95%_	*p*	AME	CI_95%_	*p*	AME	CI_95%_	*p*	AME	CI_95%_	*p*
C	–0.006	[−0.018, 0.006]	0.308	–0.009	[−0.021, 0.003]	0.127	–0.009	[−0.021, 0.003]	0.135	–0.009	[−0.021, 0.002]	0.119
ES	0.005	[−0.007, 0.017]	0.427	0.010	[−0.003, 0.022]	0.134	0.010	[−0.002, 0.023]	0.108	0.009	[−0.004, 0.021]	0.176
O	**0.011**	[0.000, 0.022]	0.041	0.008	[−0.003, 0.019]	0.145	0.007	[−0.004, 0.018]	0.222	0.007	[−0.004, 0.018]	0.219
E	0.003	[−0.009, 0.014]	0.622	0.002	[−0.010, 0.013]	0.772	0.002	[−0.010, 0.013]	0.737	0.002	[−0.009, 0.014]	0.706
A	–0.005	[−0.020, 0.010]	0.503	–0.008	[−0.022, 0.007]	0.323	–0.007	[−0.022, 0.008]	0.333	–0.008	[−0.023, 0.007]	0.308
Cognitive ability				0.000	[−0.004, 0.004]	0.985	–0.000	[−0.004, 0.004]	0.987	0.000	[−0.004, 0.004]	0.926
Parental SES				0.000	[−0.004, 0.001]	0.875	0.000	[−0.001, 0.001]	0.871	–0.000	[−0.001, 0.000]	0.759
Gender				**0.029**	[0.009, 0.050]	0.006	**0.029**	[0.009, 0.050]	0.006	**0.030**	[0.009, 0.050]	0.005
Migration background				**0.042**	[0.011, 0.072]	0.008	**0.043**	[0.012, 0.074]	0.006	**0.041**	[0.011, 0.072]	0.009
Cognitive ability × C							–0.000	[−0.005, 0.004]	0.926			
Cognitive ability × ES							–0.002	[−0.007, 0.002]	0.339			
Cognitive ability × E							0.001	[−0.004, 0.005]	0.792			
Cognitive ability × O							**0.005**	[0.001, 0.009]	0.023			
Cognitive ability × A							0.001	[−0.005, 0.007]	0.761			
SES × C										0.000	[−0.000, 0.001]	0.376
SES × ES										0.000	[−0.001, 0.001]	0.751
SES × O										**0.001**	[0.000, 0.001]	0.013
SES × E										0.000	[−0.001, 0.001]	0.941
SES × A										0.000	[−0.001, 0.001]	0.727
*Pseudo R*^2^	0.009		0.237	**0.029**		0.010	**0.037**		0.014	**0.040**		0.007

*Model I: Big Five only; Model II: Big Five and covariates; Model III: Big Five, covariates, and interaction terms between the Big Five and cognitive ability; Model IV: Big Five, covariates, and interaction terms between the Big Five and parental SES. C: Conscientiousness; ES: Emotional Stability; O: Openness; E: Extraversion: A: Agreeableness. N = 1,933. Coefficients and R^2^ significant at the p < 0.05 level are in bold type.*

From Supplementary Figure S4 it is apparent that Openness, as a positive resource to avoid forming the intentions to drop out, was more beneficial for students with low cognitive ability (0.5%; Model III) and low parental SES (0.1%) and more detrimental for students with high cognitive ability and high parental SES (Model IV).

#### Actual Dropout

The fifth success indicator was (the absence of) actual dropout from VET. As Table 7 indicates, personality alone explained 1.0% of the overall variance (*Pseudo R*^2^; Model I), with high Conscientiousness related to a 2.2% higher likelihood of not dropping out of VET, and low Agreeableness related to a 2.8% higher likelihood. Although Model II did not substantially change the relationships, it increased the overall explained variance to 1.9% (*Pseudo R*^2^). Moreover, Model II indicated that the observed association with high Conscientiousness (2.5% higher likelihood of not dropping out) was slightly higher than in Model I, and that it was incremental. The link with low Agreeableness (2.7% higher likelihood of not dropping out) remained almost the same. In addition, Model II revealed that high cognitive ability (0.7% higher likelihood) and not having a migration background (5.9% higher likelihood) were also related to not dropping out, but – after standardizing the variables – to a lesser extent than Conscientiousness (10.0%) and Agreeableness (−10.8%; cognitive ability: 8.4%).

**TABLE 7:** Average marginal effects for actual dropout regressed on the Big Five and the covariates.

Model	I			II			III			IV		
	AME	CI_95%_	*p*	AME	CI_95%_	*p*	AME	CI_95%_	*p*	AME	CI_95%_	*p*
C	**−0.022**	[−0.039, −0.004]	0.014	**−0.025**	[−0.043, −0.008]	0.004	**−0.024**	[−0.042, −0.007]	0.006	**−0.027**	[−0.044, −0.009]	0.003
ES	–0.002	[−0.019, 0.016]	0.855	0.001	[−0.017, 0.019]	0.908	0.001	[−0.018, 0.019]	0.951	0.001	[−0.017, 0.019]	0.932
O	0.012	[−0.004, 0.027]	0.143	0.012	[−0.004, 0.027]	0.147	0.011	[−0.004, 0.027]	0.159	0.009	[−0.007, 0.025]	0.253
E	0.014	[−0.002, 0.031]	0.095	0.013	[−0.004, 0.030]	0.132	0.014	[−0.003, 0.031]	0.117	0.013	[−0.004, 0.030]	0.140
A	**0.028**	[0.005, 0.051]	0.016	**0.027**	[0.004, 0.049]	0.020	**0.027**	[0.004, 0.050]	0.019	**0.029**	[0.007, 0.052]	0.011
Cognitive ability				**−0.007**	[−0.013, −0.001]	0.009	**−0.007**	[−0.012, −0.001]	0.016	**−0.007**	[−0.012, −0.002]	0.011
Parental SES				0.000	[−0.001, 0.001]	0.764	0.000	[−0.001, 0.001]	0.682	0.000	[−0.001, 0.001]	0.912
Gender				0.004	[−0.026, 0.035]	0.776	0.004	[−0.026, 0.035]	0.774	0.005	[−0.025, 0.036]	0.741
Migration background				**0.059**	[0.010, 0.109]	0.019	**0.060**	[0.011, 0.110]	0.017	**0.058**	[0.008, 0.107]	0.023
Cognitive ability × C							0.005	[−0.002, 0.011]	0.164			
Cognitive ability × ES							–0.004	[−0.011, 0.003]	0.230			
Cognitive ability × E							0.003	[−0.003, 0.010]	0.300			
Cognitive ability × O							0.001	[−0.005, 0.007]	0.779			
Cognitive ability × A							–0.002	[−0.011, 0.006]	0.600			
SES × C										0.000	[−0.001, 0.001]	0.393
SES × ES										0.000	[−0.001, 0.001]	0.817
SES × O										**0.001**	[0.000, 0.002]	0.011
SES × E										0.000	[−0.001, 0.001]	0.522
SES × A										–0.001	[−0.002, 0.000]	0.162
*Pseudo R*^2^	**0.010**		0.013	**0.019**		0.001	**0.022**		0.004	**0.026**		0.001

*Model I: Big Five only; Model II: Big Five and covariates; Model III: Big Five, covariates, and interaction terms between the Big Five and cognitive ability; Model IV: Big Five, covariates, and interaction terms between the Big Five and parental SES. C: Conscientiousness; ES: Emotional Stability; O: Openness; E: Extraversion: A: Agreeableness. N = 1,984. Coefficients and R^2^ significant at the p < 0.05 level are in bold type.*

Supplementary Figure S5 represents the same pattern as before – namely, that Openness was more detrimental for students with high parental SES, but that it compensated for low parental SES (0.1%; Model IV).

#### Final VET Grade

The sixth success indicator was the final VET grade. Table 8 indicates that personality alone explained 2.6% of the overall variance (Model I), with high Openness (*b* = 0.065) and high Extraversion (*b* = 0.068) associated with a better final VET grade. Adding the covariates in Model II increased the overall explained variance to 5.0%. In addition, the pattern showed some changes. The positive effect of Extraversion increased (*b* = 0.073) and was incremental; the positive effect of Openness vanished; and a positive effect of Conscientiousness emerged (*b* = 0.058). With regard to the covariates, Model II showed only an association with high cognitive ability (*b* = 0.035). After standardizing the independent variables, this association (*b*_std_ = 0.420) was somewhat larger than for Conscientiousness (*b*_std_ = 0.232) and Extraversion (*b*_std_ = 0.292). There were no interactive effects.

**TABLE 8:** Unstandardized regression coefficients for final VET grade regressed on the Big Five and the covariates.

Model	I			II			III			IV		
	*b*	CI_95%_	*p*	*b*	CI_95%_	*p*	*b*	CI_95%_	*p*	*b*	CI_95%_	*p*
C	0.044	[−0.011, 0.100]	0.116	**0.058**	[0.002, 0.114]	0.041	**0.058**	[0.002, 0.114]	0.042	**0.060**	[0.003, 0.116]	0.038
ES	0.038	[−0.018, 0.093]	0.183	0.031	[−0.026, 0.088]	0.287	0.032	[−0.025, 0.089]	0.267	0.030	[−0.027, 0.087]	0.305
O	**0.065**	[0.017, 0.113]	0.008	0.048	[−0.001, 0.097]	0.055	0.045	[−0.004, 0.094]	0.073	0.047	[−0.002, 0.096]	0.062
E	**0.068**	[0.016, 0.119]	0.011	**0.073**	[0.021, 0.125]	0.006	**0.075**	[0.023, 0.127]	0.005	**0.072**	[0.020, 0.124]	0.007
A	–0.041	[−0.112, 0.030]	0.256	–0.045	[−0.116, 0.025]	0.209	–0.047	[−0.118, 0.024]	0.196	–0.048	[−0.119, 0.023]	0.185
Cognitive ability				**0.035**	[0.018, 0.053]	0.000	**0.035**	[0.017, 0.053]	0.000	**0.035**	[0.017, 0.052]	0.000
Parental SES				0.002	[−0.001, 0.005]	0.121	0.002	[−0.000, 0.005]	0.114	0.002	[−0.001, 0.005]	0.127
Gender				0.026	[−0.069, 0.122]	0.588	0.029	[−0.066, 0.125]	0.546	0.027	[−0.069, 0.122]	0.583
Migration background				–0.061	[−0.252, 0.130]	0.533	–0.051	[−0.243, 0.141]	0.602	–0.066	[−0.258, 0.126]	0.499
Cognitive ability × C							–0.001	[−0.023, 0.020]	0.926			
Cognitive ability × ES							–0.006	[−0.027, 0.014]	0.540			
Cognitive ability × E							–0.009	[−0.028, 0.010]	0.371			
Cognitive ability × O							0.014	[−0.004, 0.032]	0.115			
Cognitive ability × A							–0.011	[−0.040, 0.019]	0.475			
SES × C										–0.001	[−0.004, 0.002]	0.500
SES × ES										0.000	[−0.003, 0.004]	0.914
SES × O										0.002	[−0.001, 0.005]	0.150
SES × E										0.001	[−0.002, 0.004]	0.601
SES × A										0.002	[−0.002, 0.007]	0.272
*R*^2^	**0.026**		0.001	**0.050**		0.000	**0.055**		0.000	**0.055**		0.000

*Model I: Big Five only; Model II: Big Five and covariates; Model III: Big Five, covariates, and interaction terms between the Big Five and cognitive ability; Model IV: Big Five, covariates, and interaction terms between the Big Five and parental SES. C: Conscientiousness; ES: Emotional Stability; O: Openness; E: Extraversion: A: Agreeableness. N = 813. Coefficients and R^2^ significant at the p < 0.05 level are in bold type.*

#### Satisfaction With VET

The seventh success indicator was satisfaction with VET after 1 year in a VET position. As can be seen from Table 9, a high score on both Conscientiousness (*b* = 0.129) and Emotional Stability (*b* = 0.107) was associated with the likelihood of being satisfied with VET. Personality traits explained 1.1% of the overall variance (Model I). Taking all predictor variables jointly into account, Model II, which explained 1.8% of the overall variance, revealed that the positive association with Conscientiousness increased and was incremental (*b* = 0.152). Furthermore, the positive effect of Emotional Stability disappeared, and there was also a positive effect of being male (*b* = −0.181). After the variables were standardized, Conscientiousness (*b*_std_ = 0.608) showed an even larger effect than gender.

**TABLE 9:** Unstandardized regression coefficients for satisfaction with VET after 1 year in a VET position regressed on the Big Five and the covariates.

Model	I			II			III			IV		
	*b*	CI_95%_	*p*	*b*	CI_95%_	*p*	*b*	CI_95%_	*p*	*b*	CI_95%_	*p*
C	**0.129**	[0.038, 0.221]	0.006	**0.152**	[0.060, 0.245]	0.001	**0.154**	[0.061, 0.246]	0.001	**0.155**	[0.061, 0.248]	0.001
ES	**0.107**	[0.014, 0.201]	0.024	0.076	[−0.020, 0.172]	0.122	0.078	[−0.018, 0.174]	0.110	0.077	[−0.020, 0.173]	0.119
O	–0.080	[−0.163, 0.002]	0.056	–0.066	[−0.150, 0.018]	0.123	–0.073	[−0.157, 0.011]	0.090	–0.063	[−0.148, 0.021]	0.142
E	–0.014	[−0.103, 0.076]	0.761	–0.004	[−0.094, 0.086]	0.937	–0.016	[−0.106, 0.075]	0.732	–0.002	[−0.092, 0.088]	0.964
A	0.046	[−0.074, 0.165]	0.455	0.061	[−0.058, 0.181]	0.315	0.070	[−0.050, 0.190]	0.250	0.058	[−0.063, 0.179]	0.348
Cognitive ability				0.017	[−0.013, 0.048]	0.255	0.019	[−0.011, 0.049]	0.211	0.018	[−0.012, 0.048]	0.248
Parental SES				–0.000	[−0.005, 0.004]	0.832	–0.000	[−0.005, 0.004]	0.914	–0.001	[−0.005, 0.004]	0.797
Gender				**−0.181**	[−0.345, −0.017]	0.031	**−0.174**	[−0.338, −0.010]	0.037	**−0.180**	[−0.344, −0.015]	0.032
Migration background				–0.316	[−0.676, 0.043]	0.084	–0.298	[−0.657, 0.060]	0.103	–0.319	[−0.679, 0.041]	0.082
Cognitive ability × C							0.014	[−0.022, 0.050]	0.450			
Cognitive ability × ES							–0.013	[−0.049, 0.023]	0.466			
Cognitive ability × E							0.031	[−0.003, 0.066]	0.075			
Cognitive ability × O							0.019	[−0.013, 0.051]	0.251			
Cognitive ability × A							**−0.068**	[−0.116, −0.020]	0.005			
SES × C										–0.000	[−0.005, 0.005]	0.951
SES × ES										–0.000	[−0.006, 0.005]	0.916
SES × O										–0.003	[−0.008, 0.002]	0.237
SES × E										–0.001	[−0.006, 0.004]	0.673
SES × A										0.002	[−0.004, 0.009]	0.498
*R*^2^	**0.011**		0.004	**0.018**		0.001	**0.026**		0.000	**0.019**		0.011

*Model I: Big Five only; Model II: Big Five and covariates; Model III: Big Five, covariates, and interaction terms between the Big Five and cognitive ability; Model IV: Big Five, covariates, and interaction terms between the Big Five and parental SES. C: Conscientiousness; ES: Emotional Stability; O: Openness; E: Extraversion: A: Agreeableness. N = 1,528. Coefficients and R^2^ significant at the p < 0.05 level are in bold type.*

Supplementary Figure S6 illustrates that Agreeableness compensated for low cognitive ability (*b* = −0.068; Model III).

## Discussion

The objective of the present paper was to examine whether personality contributes to success in the transition from school to VET in Germany. For this purpose, we investigated whether the Big Five personality traits had incremental associations with transition success above and beyond sociodemographic characteristics (parental SES, gender, and migration background) and cognitive ability. We defined seven indicators of transition success: obtaining a VET position, number of acceptances for VET positions, starting a VET position, (absence of) dropout intentions, (absence of) actual dropout, final VET grade, and satisfaction with VET. Moreover, we explored possible interactions of the Big Five traits with parental SES and cognitive ability.

### Additive Effects

Our findings suggest that several of the Big Five personality traits incrementally predicted at least 1 of the indicators of transition success over and above sociodemographic characteristics and cognitive ability. Among the Big Five, Conscientiousness had the most consistent positive associations with transition success. Effect sizes were small – but often as large as, or larger than, those of some of the established predictors of transition success, namely, cognitive ability and parental SES. Conscientiousness showed the most robust (incremental) predictive power for six of the seven transition success indicators: obtaining a VET position, number of acceptances for VET positions, starting a VET position, actual dropout, final VET grade, and satisfaction with VET (−0.025 ≤ AME ≤ –0.053; −0.058 ≤ *b* ≤ –0.152). This is in line with a plethora of other studies that have identified Conscientiousness as the most robust and potent predictor among the Big Five traits of educational achievement and attainment as well as career success (e.g., John et al., 1994; Poropat, 2009; Spengler et al., 2013, 2016; Woods et al., 2013; Lechner et al., 2017; Vedel and Poropat, 2017; Bergold and Steinmayr, 2018). In line with previous evidence showing links between Conscientiousness and better grades/GPA (Wintre and Sugar, 2000; Lievens et al., 2002; Trapmann et al., 2007; Poropat, 2009; Richardson et al., 2012; McAbee and Oswald, 2013; Spengler et al., 2013; Libbrecht et al., 2014; Vedel, 2014; Diedrich et al., 2018; Brandt et al., 2020) as well as satisfaction with life, work, and VET (Roberts et al., 2003; Rammstedt et al., 2017; Diedrich et al., 2018), we could support this association for almost all of our transition success indicators. A conceivable explanation is that, because of consistent performance effort during the entire vocational training period and a sense of duty and diligence, a conscientious person tends to achieve better grades, tends to be more satisfied with VET, and tends to be less likely to drop out. Furthermore, Conscientiousness manifests itself in the application behavior (in terms of the number and type of applications) and is a criterion in the selection process, thereby increasing the likelihood of obtaining a VET position and a higher number of acceptances.

The other Big Five traits had weaker and more inconsistent main effects. Extraversion (AME = 0.017; *b* = 0.073) and Agreeableness (AME = 0.027) also contributed incrementally to the prediction of transition success, whereas Openness and Emotional Stability had no incremental effects on transition success. Specifically, Extraversion predicted the final VET grade. This is in line with Wintre and Sugar (2000), who found Extraversion to be a predictor of GPA at university. Extraversion was also related to a higher likelihood of obtaining a VET position (but see Rammstedt et al., 2017, who reported a negative relation between Extraversion and the highest level of education). More extraverted students may have an advantage in obtaining an acceptance for a VET position because they are more socially connected and have more of the relevant “weak ties” (Granovetter, 1977, i.e., acquaintances compared to close friends or family members) than more introverted students. In addition, Extraversion is likely to manifest itself in the form of assertiveness in the application procedure, emboldening students to submit a greater number of unsolicited applications and to approach potential employers to inquire about vacant apprenticeship positions.

Agreeableness predicted a higher likelihood of dropping out of VET, a transition success indicator that has not been investigated to date in previous research. Our finding is in line with Lechner et al. (2017) and Brandt et al. (2020), who found negative associations between high Agreeableness and school performance using the same NEPS data. However, other studies based on other (typically much longer Big Five inventories) have reported positive associations between high Agreeableness and related outcome variables – namely, educational attainment (Shanahan et al., 2014a), sales performance and performance growth (Thoresen et al., 2004), and GPA/grades (Poropat, 2009; Richardson et al., 2012; Vedel, 2014). A possible – albeit speculative – explanation for this divergence is that different facets of Agreeableness may relate differently to different success outcomes. The BFI-10+1 measure of Agreeableness focuses mainly on the trust and compliance facets of this construct, but may not fully capture other facets that might foster success. Future research using longer Agreeableness scales – ideally scales that allow for facet-level analyses – is needed to address this question.

Some effects of individual personality traits disappeared after controlling for sociodemographic characteristics and cognitive ability. This was the case mainly with Emotional Stability and Openness, the 2 personality traits that were found to have no additive effects on transition success. Without controlling for the covariates, high Emotional Stability was positively related to starting a VET position and satisfaction with VET, and high Openness was positively associated with the final VET grade and negatively associated with the intentions to drop out. Although the very limited role of Emotional Stability contradicts our expectations, it is in line with recent large-scale findings on the Big Five as predictors of educational achievement (Lechner et al., 2017; Brandt et al., 2020) and with Poropat’s (2009) meta-analysis of personality–academic performance relationships based on the five-factor model. The prominent role of Openness in educational success suggested by this previous research was not borne out by our analyses with regard to transition to VET. A possible explanation for this is that Openness-related behaviors such as being intellectually curious or pursuing creative interests are simply not as relevant for the specific transition success outcomes that we investigated (e.g., number of acceptances for VET positions, dropout) than for more traditional indicators of academic success such as grades or test scores.

Among the covariates, migration background (as measured by the proxy of having German as a mother tongue) proved to be the most important predictor of transition success, showing significant relationships with four of the seven success indicators. However, only in 2 cases the effects of migration background were larger than that of the Big Five personality traits. Gender and cognitive ability also had significant associations with four of the seven success indicators, but to a lesser extent than migration background. The effect sizes of both gender and cognitive ability were smaller than those of the personality traits on three outcomes and larger on 1 outcome. Interestingly, parental SES was related to only 1 transition success indicator (starting a VET position), but with a smaller effect size than that of the significant personality trait Conscientiousness. Conscientiousness was more consistently related to our indicators of transition success than the established predictors. In detail, Conscientiousness was related to six of the success indicators, that is, to 2 indicators more than migration background, gender, and cognitive ability, and to five indicators more than parental SES. Despite their individually small effect sizes, the joint contribution of the Big Five personality traits in the prediction of transition success emerged as more robust than parental SES, cognitive ability, gender, and migration background.

As a consequence of the mostly small effect sizes, the overall explained variance – although significant – was not very high for any of the seven success indicators. However, this is in line with several previous investigations on relationships between the Big Five and educational or career outcomes (e.g., Rammstedt et al., 2016; Bergold and Steinmayr, 2018). Possible explanations are that almost everyone who applied for an apprenticeship got an acceptance, and that the generally small differences in the outcomes inevitably led to small variance.

### Interactive Effects

In addition to these additive effects, we explored possible interactive effects in order to further understand how personality traits might contribute to transition success. Specifically, we explored whether personality traits moderate the association with transition success of cognitive ability and parental SES.

Our exploratory findings also offer tentative support for the idea that personality traits may moderate the effects of parental SES and cognitive ability on transition success (i.e., interactive effects). Even though we found few interactive effects overall, 2 major traits showed some systematic patterns of moderation effects: Openness and Agreeableness. Openness primarily moderated the associations of parental SES with several success indicators (AME = 0.001; *b* = 0.006), whereas Agreeableness moderated solely the associations of cognitive ability with various success indicators (AME = −0.008; −0.068 ≤ *b* ≤ −0.056).

The interactive effects were mostly compensatory in nature, suggesting that personality traits can partly compensate for background disadvantages (e.g., Shanahan et al., 2014a; Damian et al., 2015; Kaiser and Schneickert, 2016; Ayoub et al., 2018), as resource substitution theory (Mirowsky and Ross, 2003) would predict. For example, high Agreeableness compensated for low cognitive ability (in predicting the number of acceptances for VET positions, satisfaction with VET), and high Openness compensated for low parental SES (in predicting the intentions not to drop out of VET, actually not dropping out of VET). The latter finding is in line with previous studies that found the same pattern, namely, compensatory interactive effects between high Openness and low parental SES in predicting educational attainment and achievement (Shanahan et al., 2014a; Kaiser and Schneickert, 2016; Ayoub et al., 2018).

Other interactions appeared to be synergistic, rather than compensatory, in nature, thus resembling the Matthew effect (Damian et al., 2015), which means that personality traits relevant to success benefited especially those who were already advantaged in terms of cognitive ability or parental SES. For example, students with high cognitive ability benefited the most from low Agreeableness (in predicting obtaining a VET position) and students with high SES benefited the most from high Openness (in predicting the number of acceptances). The latter effect is in line with Kaiser and Schneickert (2016) who examined success in primary school.

### Limitations and Directions for Future Research

The present study is among the first to address the role of personality in predicting successful educational transitions. Despite the advances we made, several limitations should be noted. First, and most importantly, although we aimed to identify causal effects of the Big Five by including several control variables and ensuring a correct temporal ordering of predictors and outcomes, unobserved third variables may have led to spurious effects. Thus, although plausible, the associations we found cannot be interpreted as causal. Experimental or quasi-experimental designs could help to overcome this limitation.

Second, only a short scale with 11 items was available to measure the Big Five personality traits. Although the short scale BFI-10 (+1; Rammstedt and John, 2007) has a relatively high predictive validity compared to longer scales (e.g., Thalmayer et al., 2011), the effect sizes we found are likely to be conservative because the BFI-10+1’s lower reliability compared to longer scales may attenuate effect sizes, and because, with 2 (or three) items per trait, the BFI-10+1 depicts each individual trait less broadly. However, the narrower operationalization may sometimes lead to higher associations with external criteria if only certain facets of each personality trait are covered that are more predictive than the dimension as a whole (Thalmayer et al., 2011). Research using longer scales – ideally scales that allow for facet-level analyses – could provide a more robust and fine-grained picture of how personality contributes to transition success.

Third, because NEPS only provides a short test of students’ cognitive ability, the internal consistency of that was relatively low in the present sample (α = 0.66). The limited reliability of the test means that, though we found significant associations between cognitive ability and some of our success indicators, the importance of student’s cognitive ability for transition success is likely to have been underestimated in the present study.

Fourth, we assumed the specific mechanisms of the Big Five traits (resource vs. selection criterion) only theoretically, and could not test them directly. Future studies are needed to reveal the mediators for the Big Five’s effects on transition outcomes.

Fifth, all seven success indicators were self-reports. Therefore, the answers on these questions could be biased by common method bias and/or socially desirable responding.

Sixth, with the available dataset, it was only possible to analyze the transition of school-leavers from *Hauptschulen* (lower secondary schools providing a basic secondary education) and *Realschulen* (intermediate secondary schools) in Germany applying for a VET position. Although we expect a similar pattern for *Gymnasium* (academically oriented secondary schools) graduates (*Abiturienten*) who apply for a VET position or for tertiary education, we cannot make generalizable predictions at this point in time. Thus, future research is needed to establish whether the present findings also apply to other educational transitions and to education systems in other national and institutional contexts.

Seventh, our tests of interactive effects were purely exploratory, we tested multiple outcome variables, and the effects did not appear consistent across all outcomes. Thus, these interactive effects should only be seen as a call for future research replicating these results and deeper investigating the causal mechanism of these effects.

## Conclusion

The present study contributes to our understanding of educational transitions by identifying Big Five personality traits as a hitherto underappreciated source of individual differences in transition success as captured by a broad range of success indicators. Our results demonstrate that several of the Big Five traits incrementally predict the successful mastery of the transition from school to VET over and above cognitive ability, parental SES, gender, and migration background. Among the variables in the model (the Big Five and the covariates), Conscientiousness proved to be the most robust (incremental) predictor of almost all the success indicators. The other Big Five traits had several additive – albeit less pervasive – associations with transition success. In addition to these additive effects, we also found evidence that personality can moderate the effects of cognitive ability and parental SES on educational transitions, and that this interaction can be both compensatory and synergistic in nature. Future research should replicate and extend these findings and generalize them to other educational transitions and education systems.

The individual effect sizes for each individual Big Five trait and success indicator were mostly small. At the same time, these effect sizes rivaled or even surpassed those of cognitive ability, parental SES, gender, and migration background. Note that these characteristics are traditionally considered to be important determinants of the success of educational transitions. Moreover, considering the combined effects of all Big Five traits on all seven success indicators, we submit that the role of personality in transition success is non-negligible and deserves greater attention in research on school-to-work transition.

Because personality traits are more malleable than sociodemographic characteristics, and hence more amenable to targeted interventions, our results also have potential practical applications. Conscientiousness and its behavioral manifestations (e.g., writing flawless VET applications and submitting them in time), for instance, could be a possible target for interventions to promote this particular personality trait and to provide specific training for those who are low in this trait, with the aim to obtain better coping strategies for educational transitions.

In sum, the findings gained from the present investigation might be of interest to educational research and policy alike. Future research could concentrate on replicating and expanding these findings. In our view, it would be particularly important to cast light on the possible mediating mechanisms linking personality to transition success. Doing so will help clarify the causes of unequal educational opportunities and make it possible to intervene purposefully.

## Data Availability Statement

The datasets analyzed in this article are not publicly available. Requests to access the datasets should be directed to fdz@lifbi.de. All data used in this study came from Starting Cohort 4 of the German National Educational Panel Study (NEPS; Blossfeld and Roßbach, 2011; doi: 10.5157/NEPS:SC4:9.1.0). From 2008 to 2013, NEPS data were collected as part of the Framework Program for the Promotion of Empirical Educational Research funded by the German Federal Ministry of Education and Research (BMBF). Since 2014, NEPS has been carried out by the Leibniz Institute for Educational Trajectories (LIfBi) at the University of Bamberg in cooperation with a nationwide network. NEPS data and documentation are provided to researchers in the form of scientific use files. Access to the data is subject to conclusion of a data use agreement (for details, visit: https://www.neps-data.de/data-center/data-access/data-use-agreements.aspx).

## Ethics Statement

The studies involving human participants were reviewed and approved by the German Federal Commissioner for Data Protection and Freedom of Information (BfDI) and in coordination with the German Standing Conference of the Ministers of Education and Cultural Affairs (KMK) and – in the case of surveys at schools – with the Educational Ministries of the respective Federal States. All data collection procedures, instruments, and documents were checked by the data protection unit of the Leibniz Institute for Educational Trajectories (LIfBi). The necessary steps are taken to protect participants’ confidentiality according to national and international regulations of data security. Participation in the NEPS study is voluntary and based on the informed consent of participants. This consent to participate in the NEPS study can be revoked at any time. Written informed consent to participate in this study was provided by the participants’ legal guardian/next of kin.

## Author Contributions

DN, DD, and CL contributed to the conception and the design of the study and edited the manuscript in several rounds. DN prepared the data, performed the statistical analysis, visualized the figures and tables, wrote the first draft of the manuscript, and conducted the revision of the manuscript. CL supervised. DD, MS, and CL reviewed and commented on the manuscript in several rounds. All authors read and approved the submitted and the final version.

## Conflict of Interest

The authors declare that the research was conducted in the absence of any commercial or financial relationships that could be construed as a potential conflict of interest.

## Footnotes

1 Between 2011 and 2014, on average 17% graduated from *Hauptschule*, 53% from *Realschule*, and 30% from *Gymnasium*. On average 98% graduates from *Hauptschule* started either a VET or a prevocational training program and less than 2% continued schooling for acquiring a university entrance qualification. On average 46% graduates from *Realschule* started either a VET or a prevocational training program and 54% continued schooling for acquiring a university entrance qualification. On average 19% graduates from *Gymnasium* started a VET and 81% went to college/university (Statistisches Bundesamt, 2013, 2014a, b, 2015). As the present study focused on VET-bound students on a vocational track graduating after 9th and 10th grade and entering VET positions, a brief description of the German dual education system, a system before labor market entry (i.e., of initial vocational training; Protsch and Solga, 2016), is needed. VET combines a on average 3-year company-based training in a specific occupation or trade, such as banker, geriatric nurse, plumbing and heating installer, or baker, with a 2-day-per-week school-based education in occupation-related subjects, such as accounting, hygiene, or medical engineering (e.g., Heckhausen and Tomasik, 2002). In some dual educational structures, for example, a 2-week training in a company alternates with a 2-week school education. Not all VET programs are eligible for all graduates, that is, some require an intermediate school-leaving degree, such as mechatronics technicians, industrial mechanics, or management assistants in wholesale, and others even an university entrance diploma (*Abitur*), such as bank clerks, insurance clerks, or IT specialists (Protsch and Solga, 2016). As a consequence, “access to the different occupations offered in the apprenticeship system is itself highly stratified by school attainment” (Protsch and Solga, 2016, p. 645).
