# Supplementary material for: Big Five Personality Traits Predict Successful Transitions From School to Vocational Education and Training: A Large-Scale Study
Source: Front Psychol. 2020 Jul 31;11:1827. doi: 10.3389/fpsyg.2020.01827 (PMC7438771; doi:10.3389/fpsyg.2020.01827)
Supplement: Supplementary file 1 [file Data_Sheet_1.PDF]

Supplementary Material

a.

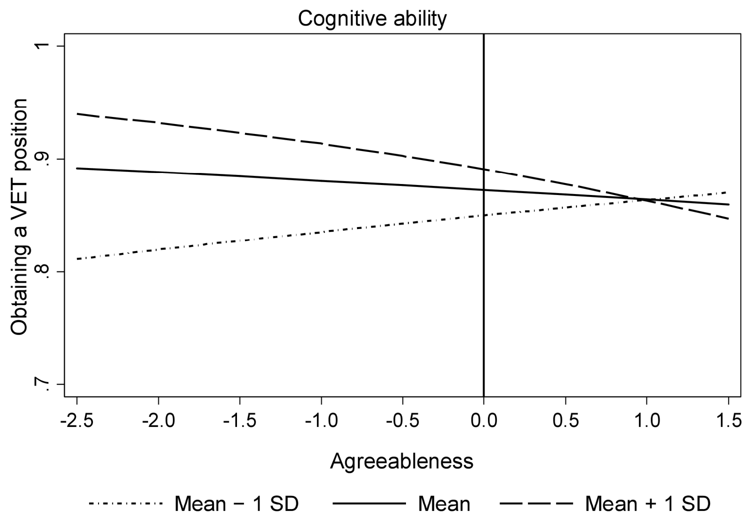

b.

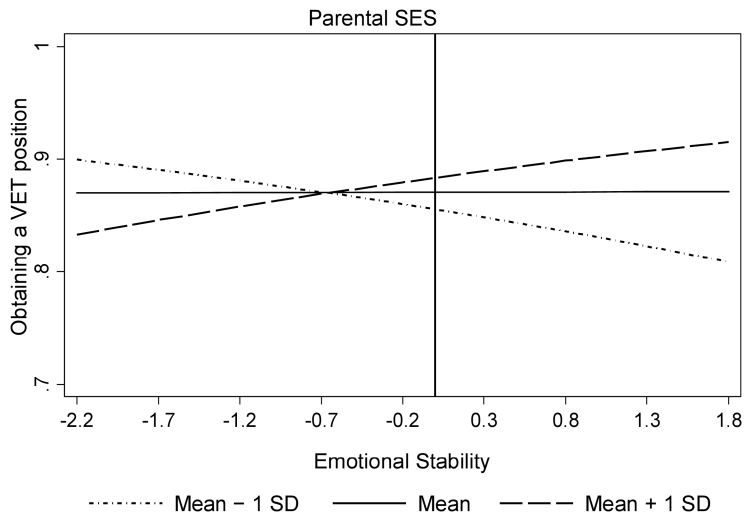

Supplementary Figure S1. Interactive associations between different Big Five personality traits and different covariates for the success indicator "obtaining a VET position." $N = 3{,}276$ (a–b). The vertical line represents the mean value. All variables are centered. All interactions are significant.

a.

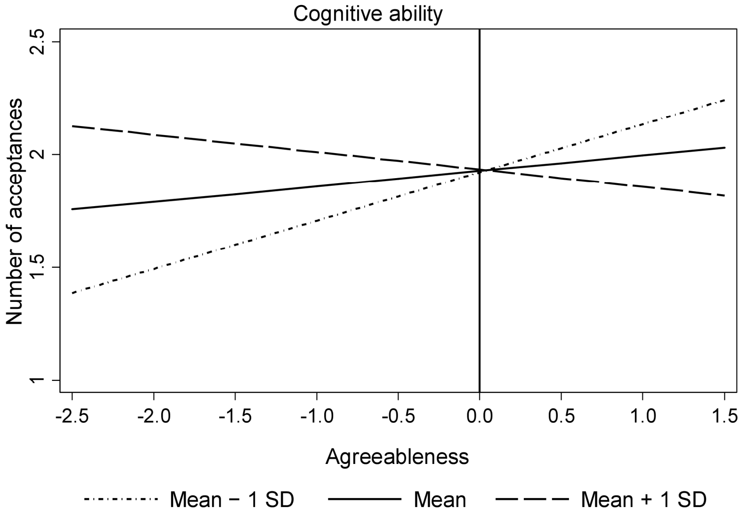

b.

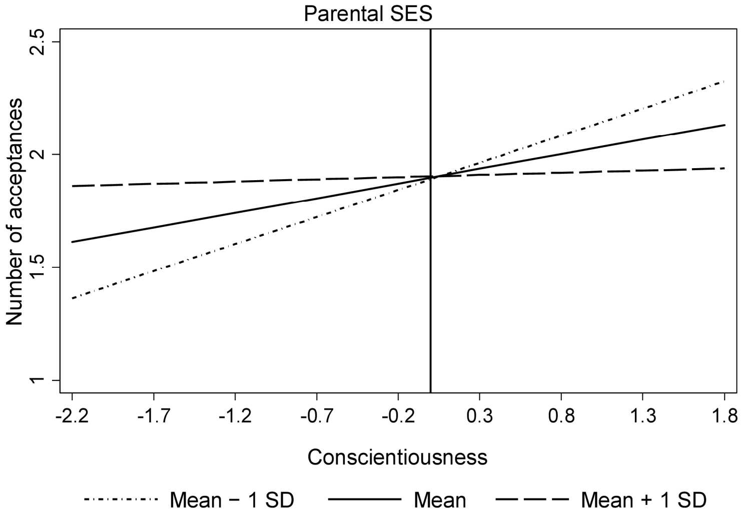

c.

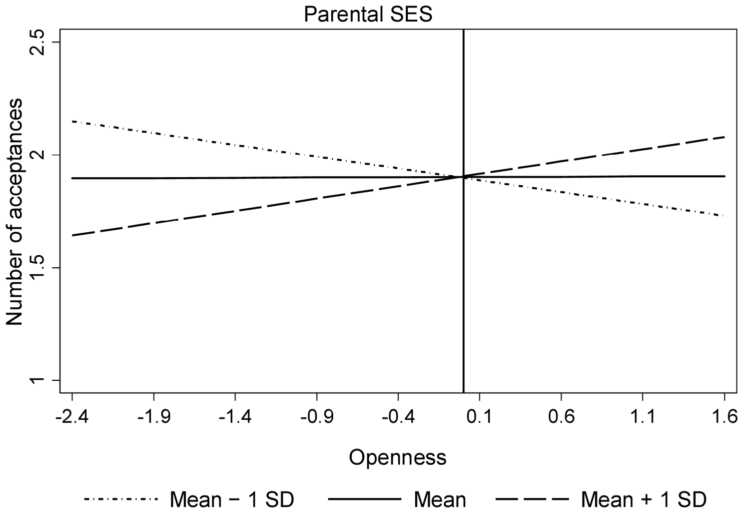

Supplementary Figure S2. Interactive associations between different Big Five personality traits and different covariates for the success indicator "number of acceptances for VET positions." $N = 2{,}606$ (a–b). The vertical line represents the mean value. All variables are centered. All interactions are significant.

a.

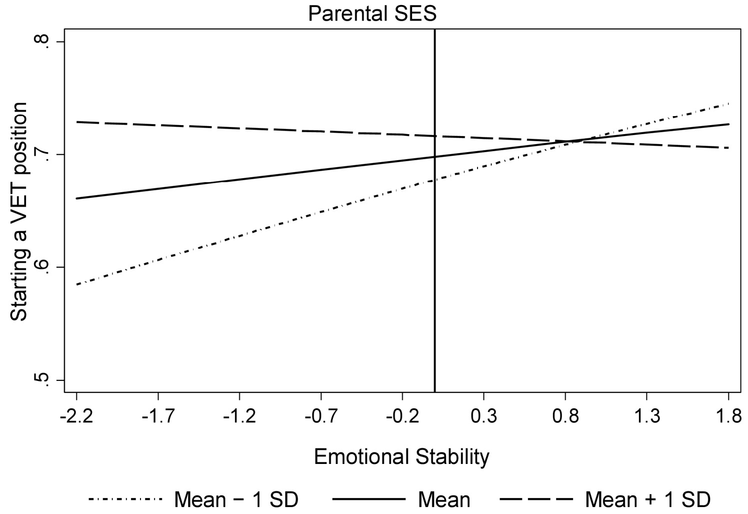

b.

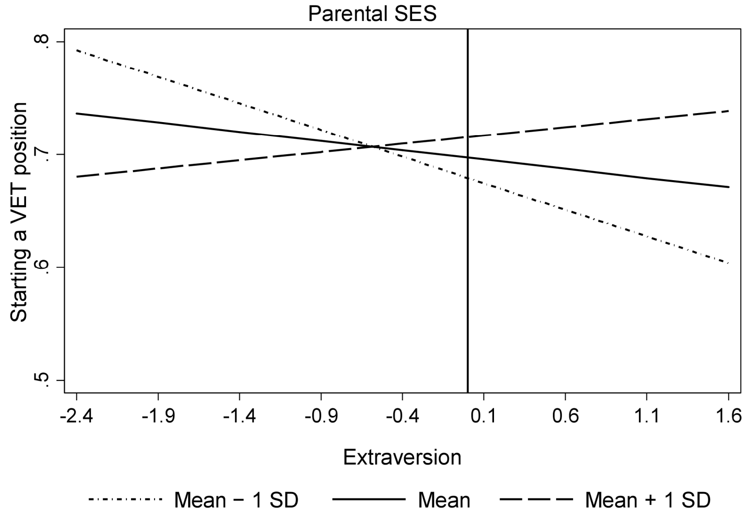

Supplementary Figure S3. Interactive associations between different Big Five personality traits and parental SES for the success indicator “starting a VET position.” $N = 2{,}846$ (a–b). The vertical line represents the mean value. All variables are centered. All interactions are significant.

a.

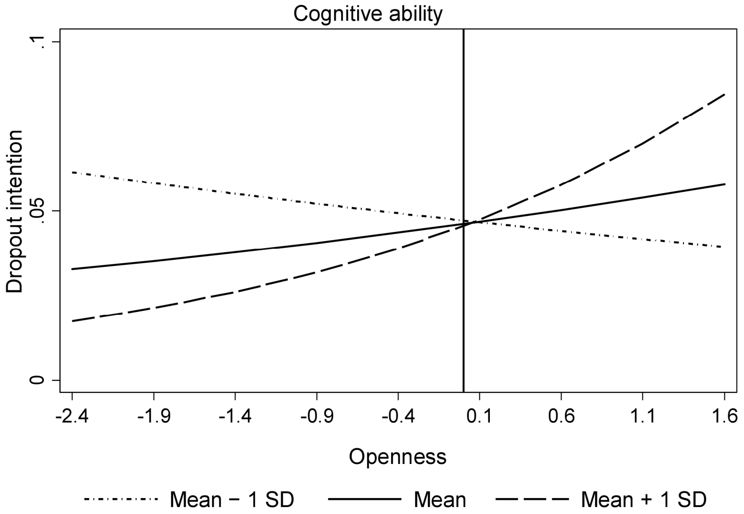

b.

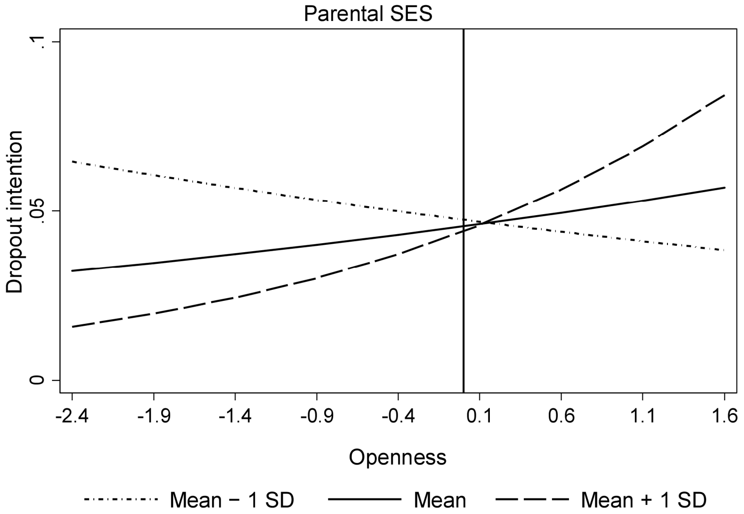

Supplementary Figure S4. Interactive associations between the Big Five personality trait Openness and different covariates for the success indicator “dropout intention.” $N = 1{,}933$ (a–b). The vertical line represents the mean value. All variables are centered. All interactions are significant.

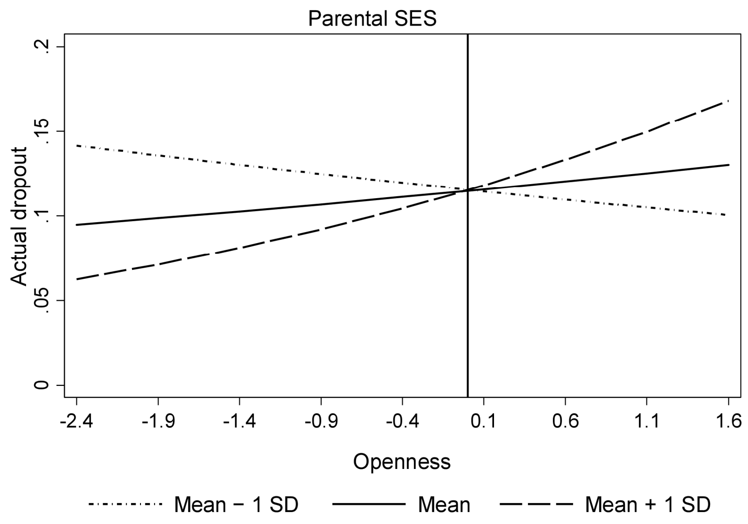

Supplementary Figure S5. Interactive associations between the Big Five personality trait Openness and parental SES for the success indicator "actual dropout." $N = 1{,}984$. The vertical line represents the mean value. All variables are centered. All interactions are significant.

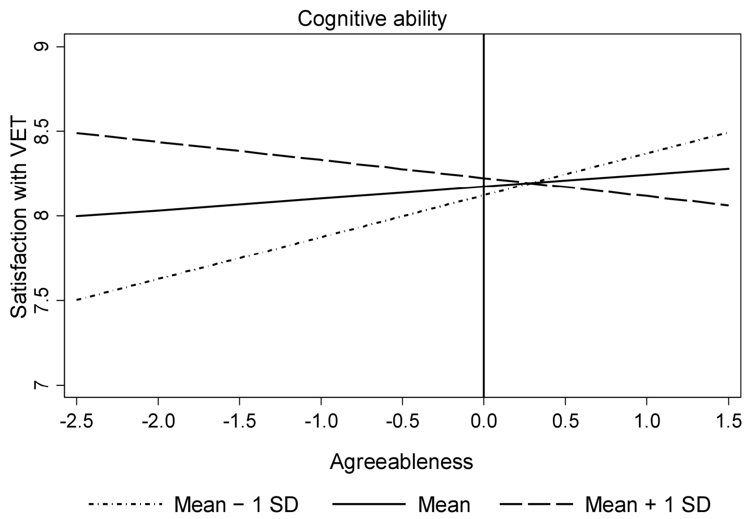

Supplementary Figure S6. Interactive association between the Big Five personality trait Agreeableness and cognitive ability for the success indicator "satisfaction with VET." $N = 1{,}528$. The vertical line represents the mean value. All variables are centered. The interaction is significant.
